# Is System x_c_^−^ a Suitable Target for Tumour Detection and Response Assessment with Imaging?

**DOI:** 10.3390/cancers15235573

**Published:** 2023-11-24

**Authors:** Amy R. Sharkey, Timothy H. Witney, Gary J. R. Cook

**Affiliations:** 1 School of Biomedical Engineering and Imaging Sciences, King’s College London, St. Thomas’ Hospital, London SE1 7EH, UK; 2 King’s College London and Guy’s and St. Thomas’ PET Centre, St. Thomas’ Hospital, London SE1 7EH, UK

**Keywords:** system x_c_^−^, redox status, response assessment, treatment resistance, treatment response

## Abstract

**Simple Summary:**

The expression of the cysteine–glutamate cotransporter, system x_c_^−^, is increased in cancer cells across many cancer types. Imaging system x_c_^−^ provides new insights into tumour behaviour. The radiotracer (4S)-4-(3-[^18^F]Fluoropropyl)-L-glutamic acid (^18^F-FSPG) is specifically transported by system x_c_^−^, allowing for a non-invasive method of measuring this transporter’s activity. This review summarises the data available on the use of ^18^F-FSPG in human cancer patients, exploring its advantages and disadvantages, and suggests possible future uses of ^18^F-FSPG in the assessment of early treatment response and treatment resistance.

**Abstract:**

System x_c_^−^ is upregulated in cancer cells and can be imaged using novel radiotracers, most commonly with (4S)-4-(3-[^18^F]fluoropropyl)-L-glutamic acid (^18^F-FSPG). The aim of this review was to summarise the use of ^18^F-FSPG in humans, explore the benefits and limitations of ^18^F-FSPG, and assess the potential for further use of ^18^F-FSPG in cancer patients. To date, ten papers have described the use of ^18^F-FSPG in human cancers. These studies involved small numbers of patients (range 1–26) and assessed the use of ^18^F-FSPG as a general oncological diagnostic agent across different cancer types. These clinical trials were contrasting in their findings, limiting the scope of ^18^F-FSPG PET/CT as a purely diagnostic agent, primarily due to heterogeneity of ^18^F-FSPG retention both between cancer types and patients. Despite these limitations, a potential further application for ^18^F-FSPG is in the assessment of early treatment response and prediction of treatment resistance. Animal models of cancer have shown that changes in ^18^F-FSPG retention following effective therapy precede glycolytic changes, as indicated by ^18^F-FDG, and changes in tumour volume, as measured by CT. If these results could be replicated in human clinical trials, imaging with ^18^F-FSPG PET/CT would offer an exciting route towards addressing the currently unmet clinical needs of treatment resistance prediction and early imaging assessment of therapy response.

## 1. Introduction

Accurate tumour detection, prediction of treatment resistance, and early response assessment represent three crucial needs in cancer care. It is now well recognised that simple metrics of changes in tumour size are an insensitive measure of tumour response and inappropriate for many targeted and biologic therapies. Although tumour detection and therapy response has been revolutionised with the advent of ^18^F-2-fluoro-2-deoxy-D-glucose (^18^F-FDG) PET/CT imaging, the success of ^18^F-FDG is not consistent across all tumour types, and alternative novel radiotracers may hold the key to tumour detection in cancers with low ^18^F-FDG uptake or where the ^18^F-FDG uptake is obscured by high physiological retention in healthy tissues. Imaging of the redox status via imaging of system x_c_^−^ [1] may provide new insights into tumour behaviour, which could facilitate not only tumour detection but also early evaluation of response assessment and treatment resistance.

### 1.1. Redox Status

Cellular redox status is the balance between oxidants and antioxidants within a cell. This balance is known to regulate biological responses and events, including the expression of multiple gene-encoded proteins affecting cell death and survival [2]. An imbalance between the production of oxidants, primarily reactive oxygen species, and their removal by antioxidant defence systems causes oxidative stress, with resulting deleterious effects.

Cellular redox status is particularly important in tumour cells, which require an adequate supply of nutrients to meet increased anabolic and energetic needs while maintaining appropriate redox balance for growth, proliferation, and survival. Elevated metabolic activity, coupled with high growth and often insufficient vascular supply, results in increased reactive oxygen species, leading to high levels of oxidative stress, which can affect many redox-sensitive molecular pathways involved in cell survival [3]. To meet their increased metabolic needs, tumour cells upregulate multiple metabolic pathways [4,5]. The upregulation of the glycolytic pathways in cancer is well-established, and the use of ^18^F-FDG in the molecular imaging of tumours is routine in cancer care. However, alternative upregulated metabolic pathways in tumours, such as the glutathione (GSH) biosynthesis/redox-balancing pathway, also represent possible targets in the molecular imaging of cancer, with the potential to improve detection and molecular characterisation of tumours.

### 1.2. System x_c_^−^

**Figure 1:**
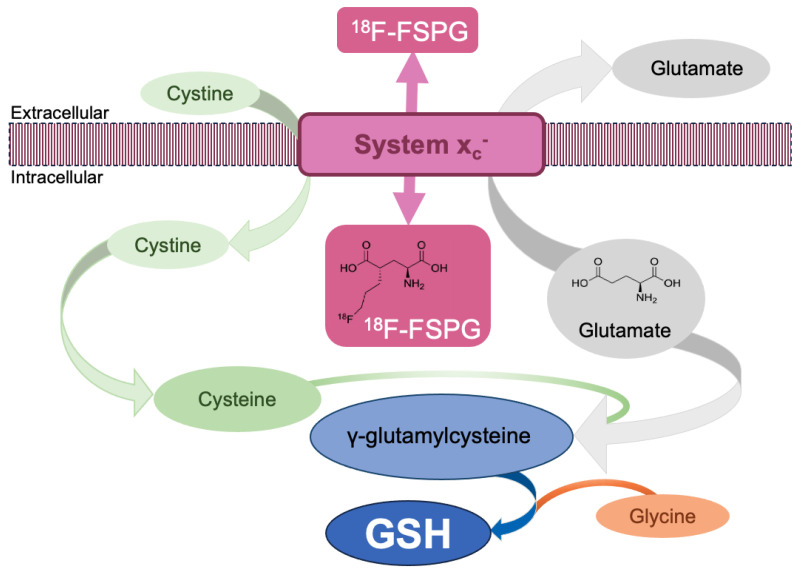
System x_c_^−^ schematic. System x_c_^−^ imports cystine in exchange for intracellular glutamate, providing a surrogate marker of GSH biosynthesis. ^18^F-FSPG is an L-glutamate derivative used as a PET biomarker to assess intracellular redox status in vivo through targeting of the system x_c_^−^ transporter.

System x_c_^−^ is a heterodimeric amino acid transporter composed of a light chain (xCT or *SLC7A11*) and a heavy chain 4F2hc. The heavy chain 4F2hc is a single transmembrane spanning glycoprotein (also called CD98hc or *SLC3A2*), necessary for correctly trafficking the transporter to the plasma membrane [6]. System x_c_^−^ is an antiporter of cystine, the oxidised form of cysteine, which is taken into the cell in exchange for glutamate. Intracellularly, cysteine is required for protein synthesis, facilitates redox-sensitive reactions through its thiol, and provides the rate-limiting substrate for GSH biosynthesis (Figure 1), the body’s most abundant antioxidant. As well as directly measuring GSH concentration in tissues, the biosynthesis of GSH can be indirectly assessed by measuring system x_c_^−^’s activity [7].

System x_c_^−^ has relatively low basal expression in most tissues but is upregulated in cells undergoing oxidative stress, such as tumour cells [8]. The role of system x_c_^−^ has been extensively investigated in animal models of cancer, confirming its key role in tumour growth and progression [7]. Importantly, inhibition of system x_c_^−^ suppresses tumour growth in animal models [9]. Furthermore, deletion of xCT in murine models of pancreatic cancer induced tumour selective ferroptosis [10], a form of programmed cell death that results from the overaccumulation of reactive oxygen species, membrane damage, and consequent inhibition of tumour growth.

### 1.3. Radiopharmaceuticals Imaging Oxidative Stress

Imaging and quantification of oxidative stress via measurement of system x_c_^−^ activity allows for the examination of cellular redox status in vivo. (4S)-4-(3-[^18^F]fluoropropyl)-L-glutamic acid (^18^F-FSPG) is a glutamate analogue which can be used as a positron emission tomography (PET) tracer to measure system x_c_^−^ activity, and specific transport of ^18^F-FSPG via system x_c_^−^ has been demonstrated in cell competition assays and xCT knock-down cells [11]. Net ^18^F-FSPG retention is a measure of both ^18^F-FSPG uptake and ^18^F-FSPG efflux across the cell membrane.

Early human trials of ^18^F-FSPG PET/CT in healthy volunteers [12] and in cancer patients have been performed, showing consistent biodistribution data with high tumour-to-background ratios in some, but not all, cancers [13,14]. No clinical trials using tracers targeting system x_c_^−^ other than ^18^F-FSPG have been published to date. However, other PET tracers targeting system x_c_^−^, ^18^F-hGTS13, (*R*)-4-(3-^18^F-fluoropropyl)-L-glutamate (^18^F-FRPG) and 18F-5-fluoro-L-aminosuberic acid (^18^F-FASu), have been recently described in preclinical models. Beinat et al. [15] compared the performance of ^18^F-hGTS13 to ^18^F-FSPG in cell culture and animal tumour models, finding excellent tumour visualisation, with high tumour-to-background ratios obtained with ^18^F-hGTS13. Greenwood et al. [16] compared ^18^F-FSPG to its enantiomer ^18^F-FRPG, finding that there was not only fast clearance and low background retention of ^18^F-FRPG but that in in vivo models, ^18^F-FRPG showed a higher percentage of parent radiotracer in tumours compared to ^18^F-FSPG, allowing for clear tumour visualisation. In addition, ^18^F-FRPG was sensitive to changes in the cellular redox status and tumour retention of ^18^F-FRPG was reduced following chemotherapy treatment in mice with ovarian tumours, suggesting that ^18^F-FRPG could allow for tumour treatment-response monitoring. Webster et al. [17] compared ^18^F-FASu to ^18^F-FDG using in vivo models of oxidative stress and found ^18^F-FASu uptake was approximately five times greater than ^18^F-FDG uptake in some tumours, suggesting that ^18^F-FASu might provide more sensitive detection than ^18^F-FDG for certain indications. ^18^F-FASu has also been directly compared with ^18^F-FSPG [18], showing that while ^18^F-FSPG had greater in vitro uptake than ^18^F-FASu in all cancer cell lines tested (prostate, glioblastoma, colorectal, ovarian, and lung), both radiotracers generated high-contrast PET images in mice with glioblastoma and lung tumours. Overall, these findings suggest a role for system x_c_^−^ radiotracers for cancer imaging, although prospective clinical trials are required to determine if the same results will be replicated in human data.

### 1.4. Oxidative Stress and Treatment Resistance

Biochemical antioxidant mechanisms play a critical role in the development of acquired treatment resistance in cancer. Cancer treatments, such as chemotherapy, have been shown to produce oxidative stress, resulting in cell death in tumours sensitive to treatment [19,20]. However, the clinical benefit of some promising cancer therapies is limited as a cohort of patients are inherently resistant and fail to respond to treatment or subsequently develop treatment resistance. Treatment resistance is frequently caused through the upregulation of GSH, which preserves cellular redox status and provides defence against the reactive oxygen species produced by therapy-induced oxidative stress [21]. The concentration of GSH is known to be dramatically increased in therapy-resistant tumours [22].

Tumours prone to redox stress may be especially vulnerable to system x_c_^−^ disruption, which is thought to play a role in GSH-based drug resistance [23]. GSH-based drug resistance has been studied preclinically, primarily in ovarian tumours, which often develop resistance to chemotherapy [24]. In human ovarian cancer, while approximately 75% of patients initially respond to platinum/paclitaxel-based chemotherapy, most patients relapse with chemotherapy-resistant disease, which results in treatment failure and is responsible for over 90% of cancer-related mortality [24]. Ex vivo studies of biopsy samples of human ovarian cancer have shown GSH levels increase up to 10-fold in tumours following the development of resistance to alkylating agents compared with the samples taken before treatment [25].

As system x_c_^−^ activity can be measured with ^18^F-FSPG PET, this type of imaging could offer a method to evaluate treatment resistance in vivo. Preclinical studies using mouse models of chemotherapy-resistant ovarian cancer [26] found that high intracellular GSH and low levels of reactive oxygen species corresponded to decreased ^18^F-FSPG cell accumulation in chemotherapy-resistant versus chemotherapy-sensitive ovarian cancer cell lines. Furthermore, chemotherapy-sensitive cells had high pre-treatment ^18^F-FSPG retention, whereas there was low intracellular ^18^F-FSPG retention in the chemotherapy-resistant cells, indicating that pre-treatment ^18^F-FSPG imaging could reveal drug-resistant disease [26].

In vivo, ^18^F-FSPG retention was 80% lower in chemotherapy-resistant tumours compared with matched chemotherapy-sensitive tumours, and treatment of drug-resistant tumours with doxorubicin resulted in no detectable changes in either tumour volume, GSH concentration, or ^18^F-FSPG retention. Similar mouse models have demonstrated tumour cell retention of ^18^F-FSPG decreases in proportion to levels of oxidative stress following treatment with a range of redox-active compounds [1].

### 1.5. Imaging Response to Therapy

Predicting treatment resistance and early response assessment are critical for personalising cancer therapy and represent a significant unmet need in cancer care. Imaging with ^18^F-FSPG may enable early assessment of response; in mouse models of ovarian cancer, a decrease in ^18^F-FSPG preceded both tumour shrinkage measured by CT and glycolytic changes assessed with ^18^F-FDG [1,26], which are the generally accepted clinical methods of assessing treatment response. Treatment response has been further assessed using ^18^F-FSPG PET/CT in mouse models of colorectal cancer, comparing known treatment-sensitive and treatment-resistant tumours [27]. In the mouse models with patient-derived treatment-sensitive xenografts, ^18^F-FSPG retention was significantly decreased from baseline at one-week post-therapy (epidermal growth factor receptor-targeted monoclonal antibody therapy, glutaminase inhibitor therapy, or combination) prior to changes in tumour volume (as measured using callipers). In contrast, ^18^F-FSPG retention was not decreased in patient-derived treatment-resistant xenografts. Although these studies have only been performed in animal models thus far, they suggest that ^18^F-FSPG PET imaging could provide a sensitive and non-invasive method of assessing early treatment response.

To date, the only clinical trials targeting system x_c_^−^ in humans have been via the use of ^18^F-FSPG. If preclinical findings demonstrating the ability of ^18^F-FSPG to predict treatment resistance and assess early treatment response could be translated to human studies, this would be extremely valuable; patient management and treatment selection could then be altered accordingly, with the possibility of improving clinical outcomes.

## 2. Imaging System x_c_^−^ Activity in Humans

### Biodistribution

**Figure 2:**
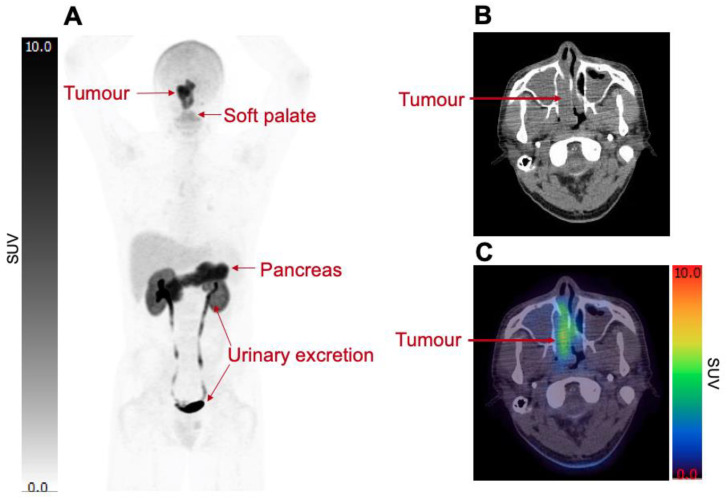
Normal biodistribution of ^18^F-FSPG. The ^18^F-FSPG imaging at 90 min p.i. (SUV range: 0–10) in a patient with a primary nasopharyngeal tumour. Coronal MIP imaging (**A**) shows normal physiological uptake of ^18^F-FSPG in the soft palate, liver, and pancreas with renal excretion, as well as pathological uptake in the primary tumour. Low background activity in the majority of organs was noted. Axial CT (**B**) and axial fused images (**C**) demonstrate the ^18^F-FSPG uptake in the primary tumour (SUV_max_ = 10.9). The Figure was created from data acquired by Park S. Y. et al. [14] and provided by Life Molecular Imaging (Berlin, Germany).

Pilot ^18^F-FSPG imaging was performed in humans, with dosimetry [28] and biodistribution [12] in healthy participants evaluated. Dosimetry calculations were undertaken in five healthy volunteers, where the effective dose for ^18^F-FSPG was 9.5 ± 1.0 mSv at an administered activity of 300 MBq, which is of similar magnitude to that of ^18^F-FDG. ^18^F-FSPG was excreted renally, and the absorbed dose was highest in the urinary bladder wall and kidneys; the effective dose was reduced to 4.5 ± 0.30 mSv (at 300 MBq administered activity) with regular bladder-voiding every 45 min [28]. Example ^18^F-FSPG imaging is shown in Figure 2, demonstrating both physiological and pathological uptakes.

^18^F-FSPG has a consistent biodistribution pattern with low background retention in most tissues. Biodistribution data were evaluated in five healthy volunteers [12], each undergoing seven PET scans, performed at 5, 15, 25, 35, 45, 150, and 240 min post-injection (p.i.). Time-activity curve analyses showed rapid clearance of the radiotracer from the blood pool and from most organs except the pancreas. ^18^F-FSPG did not pass the intact blood–brain barrier, with radiotracer retention in the brain close to zero across all time points, and lung activity was low (mean standardised uptake values (SUV_mean_) < 2) 5 min p.i., and close to zero at 45 min p.i.. As ^18^F-FSPG is renally excreted, the kidneys had the highest SUV_mean_ until 45 min p.i. (SUV_mean_ 16.3 ± 2.2), after which the SUV_mean_ fell (150 min: 4.7; 240 min: 2.3). The pancreas showed an initial increase in SUV_mean_ over the study period: at 5 min p.i., SUV_mean_ was 5.6; at 45 min, it was 8.4, decreasing to 5.3 and 3.1 at 150 and 240 min, respectively. High physiological retention of ^18^F-FSPG in the pancreas is a result of elevated expression of system x_c_^−^ compared to other tissues [29]. The pancreas has high GSH, which is essential for digestive enzyme synthesis, which may explain high ^18^F-FSPG retention in this organ [30].

## 3. Diagnostic Performance of ^18^F-FSPG in Cancer Patients

For tumour detection, ^18^F-FSPG offers some benefits over ^18^F-FDG as a consequence of low physiological retention in most organs (Figure 2), leading to higher tumour-to-background ratios in several cancer types. Multiple papers describe the utility of ^18^F-FSPG versus ^18^F-FDG in organs with high ^18^F-FDG retention. For example, in head and neck cancers, tumours close to the skull base can be obscured by physiological brain retention of ^18^F-FDG or by physiological retention of lymphoid tissue in Waldeyer’s ring. Similarly, physiological retention within the heart can potentially mask thoracic metastases. A case study of a single patient with non-small cell lung cancer (NSCLC) showed that myocardial and pericardial metastases were obscured by physiologic ^18^F-FDG cardiac retention that was revealed with ^18^F-FSPG [31], although metastases in the heart are rare. Conversely, due to high physiological retention in the pancreas and renal excretion, ^18^F-FSPG may be less useful in the detection of primary pancreatic or renal cortex tumours. Another benefit of ^18^F-FSPG is that neither resting nor fasting is required prior to its administration, whereas close control of physical activity and blood glucose levels is important prior to ^18^F-FDG injection.

^18^F-FSPG PET/CT has been evaluated in a range of different cancer types, primarily as a diagnostic modality for tumour detection. All papers state that ^18^F-FSPG was well tolerated, and there was similar biodistribution in normal organs across patients. The majority of these papers describe the use of ^18^F-FSPG PET/CT in a relatively small number of cancer patients (range 1–26) across a range of different cancers. A major limitation of all studies to date is that the relatively small numbers limit the inferences and conclusions that can be drawn. A summary of the papers evaluating ^18^F-FSPG PET/CT in cancer patients is provided in Table 1.

### 3.1. Comparison of ^18^F-FSPG PET/CT Imaging with ^18^F-FDG PET/CT

**Figure 3:**
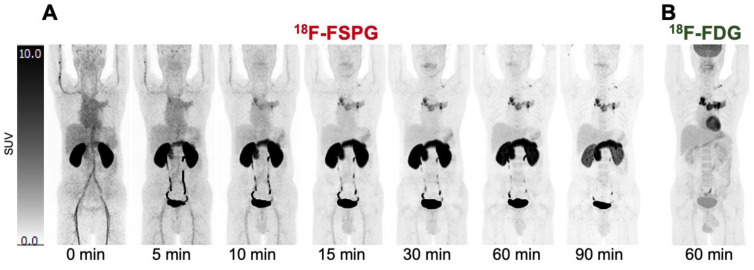
^18^F-FSPG vs. ^18^F-FDG imaging. ^18^F-FSPG scans (SUV range: 0–10) in a patient with metastatic NSCLC at intervals up to 90 min p.i. (**A**), demonstrating rapid accumulation in the primary and mediastinal metastatic deposits, comparable to a standard 60 min p.i. ^18^F-FDG PET scan (**B**). The Figure was created from data acquired by Baek S. et al. [37] and provided by Life Molecular Imaging.

The performance of ^18^F-FSPG PET/CT has been compared to standard-of-care imaging, most often ^18^F-FDG PET/CT (Figure 3). The relative diagnostic performance of ^18^F-FSPG PET/CT appears dependent on the cancer subtype.

In pancreatic cancer, ^18^F-FSPG PET proved superior to ^18^F-FDG PET in detecting metastases despite similar tumour-to-background ratios on positive scans (^18^F-FSPG: 4.2 ± 4.3 vs. ^18^F-FDG: 3.6 ± 3.0) [34]. In a patient-based analysis, ^18^F-FSPG PET had higher sensitivity (95% vs. 90%), specificity (100% vs. 66.7%), and diagnostic accuracy (95.7% vs. 90%) than ^18^F-FDG PET. In a lesion-based analysis in the same cohort, ^18^F-FSPG PET identified significantly more pancreatic cancer metastases, especially in the liver, than ^18^F-FDG PET (109 vs. 95; *p* < 0.01). Similarly, good performance was described in hepatocellular carcinoma, in which ^18^F-FSPG PET detected lesions in 5/5 patients, whereas ^18^F-FDG detected lesions in only 3/5 patients with hepatocellular carcinoma (HCC) [13]. These results may be partly related to the fact that the SUV_mean_ of normal liver is generally lower with ^18^F-FSPG than that of ^18^F-FDG, both in normal volunteers (SUV_mean_ at 60 min p.i. of 1.5 ± 0.4 with ^18^F-FSPG vs. 2.2 ± 0.4 with ^18^F-FDG) and in patients with HCC (SUV_mean_ at 60 min p.i.: 1.3 ± 0.3 with ^18^F-FSPG vs. 1.9 ± 0.2 with ^18^F-FDG) [13]. Both tracers have similar tumour uptake (SUV_max_ for ^18^F-FSPG: 4.7 ± 3.2 vs. ^18^F-FDG: 6.1 ± 2.9), suggesting that the significantly lower SUV_mean_ (*p* < 0.05) of the normal liver with ^18^F-FSPG imaging may increase lesion detection. ^18^F-FSPG imaging could be of clinical value in HCC given the relatively poor sensitivity of ^18^F-FDG PET/CT in the detection of well-differentiated HCC [39,40], where alternative tracers, such as ^11^C-acetate or ^11^C/^18^F-choline, have been explored [41].

**Figure 4:**
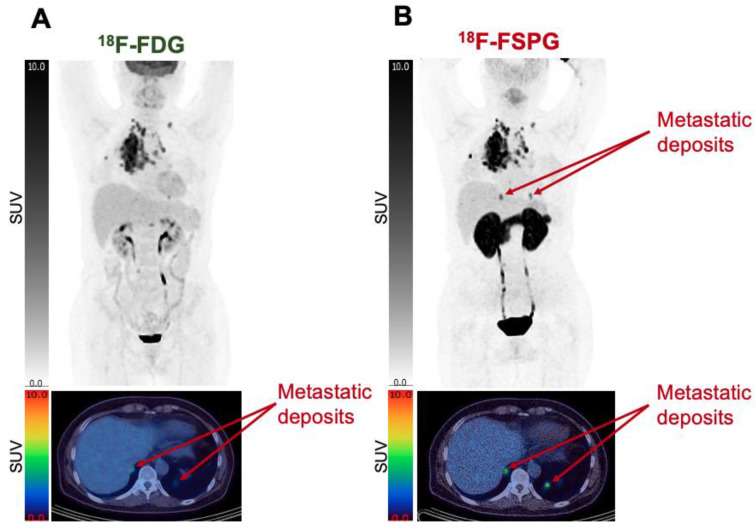
Comparative imaging shows differential uptake of ^18^F-FSPG vs. ^18^F-FDG in some metastatic deposits. ^18^F-FDG coronal MIP imaging (**A**) vs. ^18^F-FSPG coronal MIP imaging (**B**) highlights two lesions, one in the right and one in the left lower pulmonary lobes, which are easily detectable with ^18^F-FSPG but poorly discernible with ^18^F-FDG. Both images are 60 min p.i., with SUV range: 0–10. The Figure was created from data acquired by Baek S. et al. [37] and provided by Life Molecular Imaging.

In patients with NSCLC, the results were more variable. In a study of ten patients with NSCLC comparing the performances of ^18^F-FSPG and ^18^F-FDG, the tumour-to-blood pool SUV ratio was not significantly different between the two tracers, with an SUV ratio of 9.7 ± 8.6 with ^18^F-FSPG vs. 7.7 ± 4.2 with ^18^F-FDG (*p* = 0.12) [37]. ^18^F-FSPG identified all ten NSCLC lesions that were confirmed by pathology and was able to detect 59/67 (88%) of ^18^F-FDG-positive lesions (Figure 4). ^18^F-FSPG additionally detected seven further lesions that were not detected with ^18^F-FDG; this is of clinical relevance as the identification of these lesions could lead to a change in patient staging and management.

A second study reporting the performance of ^18^F-FSPG in lung pathology found a similar performance of ^18^F-FDG and ^18^F-FSPG in the evaluation of both indeterminate lung lesions and in the staging of lung cancer [38]. In benign lesions, there were minimal differences in performance; ^18^F-FSPG was negative in 6/9 benign lesions compared to 7/9 with ^18^F-FDG. The ^18^F-FSPG SUV_max_ was lower in both benign (SUV_max_: 0.7 (range: 0.4–1.0)) and malignant (SUV_max_: 1.2 (range: 1–1.7)) lesions compared to ^18^F-FDG (benign SUV_max_: 1.4 (range: 0.7–1.8), malignant (SUV_max_: 2.2 (range: 1.6–4.3)). Overall, the sensitivity of ^18^F-FSPG and ^18^F-FDG for indeterminate pulmonary nodules was 75% and 58%, the specificity was 67% and 78%, and the accuracy was 71% and 67%, respectively. In malignant lesions, ^18^F-FSPG was positive in 14/17, and ^18^F-FDG was positive in 12/17 (12 NSCLC, one recurrent metastatic renal cell carcinoma, one sarcomatoid carcinoma, one small cell carcinoma, two unknown histological subtypes). When these 17 malignant lesions were subdivided into pulmonary nodules (7–30 mm) and lung masses (>30 mm), ^18^F-FSPG was positive in 9/12 pulmonary nodules, and ^18^F-FDG was positive in 7/12 pulmonary nodules. Both tracers were positive in 5/5 lung masses. Of the two nodules that were negative with ^18^F-FDG but positive on ^18^F-FSPG, one was Stage IIB NSCLC, and the other was of unknown histological subtype but presumed malignant due to interval growth and highly atypical cells on biopsy, suggesting that imaging with ^18^F-FSPG may be useful in the detection of atypical malignancies.

In other cancer types, the diagnostic performance of ^18^F-FSPG was below that of ^18^F-FDG. In five patients with breast cancer, the SUV_max_ of tumours was significantly lower with ^18^F-FSPG vs. ^18^F-FDG (2.1 ± 1.5 vs. 14.8 ± 1.2), and ^18^F-FSPG was only able to identify 3/5 histologically confirmed breast primary breast cancers (all ductal carcinoma), and 30/73 (41%) ^18^F-FDG positive metastatic lesions [37]. This study also included ten patients with NSCLC, allowing for a direct temporaneous comparison of the performance of ^18^F-FSPG in NSCLC versus breast cancer. There were large variations in the SUV_mean_ of ^18^F-FSPG, both among individual patients and in different tumour types (SUV_mean_ NSCLC: 5.4 ± 4.8; breast: 1.8 ± 1.2 at 60 min p.i.), in keeping with differences in the underlying tumour biology and cellular redox status.

A second study comparing the performance of ^18^F-FSPG across different cancer types also revealed mixed results [14]: 100% of patients with colorectal cancer had high retention in primary tumours (average SUV_mean_ at 60 min p.i.: 5.4 ± 1.3), 80% of those with head and neck cancer (average SUV_mean_ at 60 min p.i.: 2.8 ± 2.1), but the retention in patients with non-Hodgkin’s lymphoma (NHL) was variable (average SUV_mean_ at 60 min p.i.: 1.9 ± 3.1). Five patients with NHL were included, with different subtypes (two mantle cells, one diffuse large B cell lymphoma (DLBCL), one follicular and one cutaneous T cell lymphoma). These patients showed the greatest amount of variability between the ^18^F-FSPG and ^18^F-FDG PET scans; the patients with follicular lymphoma and DLBCL had low retention of ^18^F-FSPG (essentially equivalent to the liver background), whereas they exhibited mild-to-moderate retention with ^18^F-FDG. Of the two patients with mantle cell lymphoma, one showed the lowest ^18^F-FSPG SUV values of all five NHL subjects, while the other showed the highest ^18^F-FSPG SUV values, with the ^18^F-FSPG SUV nearly double that of the comparative ^18^F-FDG SUV.

### 3.2. Comparison of ^18^F-FSPG PET/CT with Other Radiotracers Using PET/CT

Only one study compared the performance of ^18^F-FSPG to PET imaging with a tracer other than ^18^F-FDG. Kavanaugh et al. [35] examined ^18^F-FSPG retention in a cohort of 11 patients with hepatocellular carcinoma and compared ^18^F-FSPG to ^11^C-acetate PET/CT in 7/11 patients, demonstrating a 90% detection rate with ^18^F-FSPG and a 70% detection rate with ^11^C-acetate PET/CT. In tumours which were positive on both ^18^F-FSPG and ^11^C-acetate PET/CT, ^18^F-FSPG accumulation consistently resulted in significantly greater tumour-to-liver background ratios compared with ^11^C-acetate PET/CT, partially due to the low ^18^F-FSPG SUVs of background liver (range SUV_max_: 0.5–1.9). Additionally, ^18^F-FSPG PET revealed a primary hepatocellular carcinoma, which was missed on the standard-of-care imaging as it was devoid of a typical MRI enhancement pattern [35].

### 3.3. Comparison of ^18^F-FSPG PET/CT with Standard-of-Care MRI

In tumours where the standard-of-care diagnostic modality is MRI rather than ^18^F-FDG PET/CT, for example, prostate cancer, ^18^F-FSPG PET showed high tumour-to-background ratios with a relatively high tumour detection rate on a per-patient (89%) and per-lobe (87%) basis [33]. In the setting of recurrent prostate cancer, agreement with MRI was demonstrated in 7/9 patients (78%). In the assessment of extra-prostatic metastatic lesions, ^18^F-FSPG PET/CT showed agreement with standard imaging in 13/18 lesions (72%), corroborating both nodal and distant metastatic disease (bone and lung). ^18^F-FSPG PET/CT also revealed true local recurrence in a discordant case; a CT performed just prior to the ^18^F-FSPG PET/CT showed multiple foci of bony sclerosis, suspicious for osseous metastases in the context of rising prostate-specific antigen (PSA), whereas the ^18^F-FSPG PET/CT showed intense retention in only the right apex of the prostate, not in any of the bone lesions. The prostatic lesion was subsequently confirmed with MRI, and the patient underwent local brachytherapy, after which the PSA dropped to undetectable levels within three months. For many years, follow-up CT and MR scans continued to show that the bone lesions were stable, confirming their benign nature and that the ^18^F-FSPG PET was a true negative. Together, these findings suggest that ^18^F-FSPG PET/CT may have a complementary diagnostic role in some circumstances where standard-of-care imaging falls short.

### 3.4. Histopathology Comparison

There are no well-validated antibodies for xCT immunohistochemistry [42]. Not all clinical studies evaluating ^18^F-FSPG used a histopathological reference standard, and those that used varied markers to measure xCT expression, usually xCT and/or CD44 antibodies [7,9]. CD44 is a transmembrane protein and functions as a cellular adhesion molecule. A splice variant of CD44 (CD44v) interacts with and stabilises the xCT subunit and thereby promotes cystine retention for GSH biosynthesis to counteract high levels of oxidative stress [43].

The results of studies using a histopathological reference standard are mixed: in prostate cancer patients [33], ^18^F-FSPG accumulation showed only a moderate, non-statistically significant correlation (Spearman correlation coefficient *ρ* = 0.41; *p* = 0.21) with CD44 expression. In hepatocellular carcinoma, two patients with both xCT and CD44 expression had moderate or intense accumulation of ^18^F-FSPG, whereas two patients with negative CD44 expression showed mild ^18^F-FSPG retention [13]. A comparative study of five patients with breast cancer and ten patients with NSCLC [37] looked at both xCT and CD44 expression, finding a significant correlation of ^18^F-FSPG SUV_max_ at 60 min p.i. with both xCT (*p* = 0.68, *p* < 0.01) and CD44 expression (*p* = 0.77, *p* < 0.01). One reason for these mixed results could be the use of nonspecific anti-xCT antibodies, which rely on the predicted molecular weight of 55.5 kDa to identify xCT on western blots [44]. Further work is needed to produce validated antibodies for xCT immunohistochemistry to provide a more reliable histochemical reference standard for clinical studies.

### 3.5. Heterogeneity of ^18^F-FSPG Retention

As mentioned above, most studies evaluating the use of ^18^F-FSPG in human subjects with cancer compared the diagnostic performance of ^18^F-FSPG PET/CT versus ^18^F-FDG PET/CT; a consistent theme throughout these studies was the heterogeneity of ^18^F-FSPG retention. This heterogeneity may be due to differences in retention within a single tumour (intra-tumoural), differences in retention between the primary tumour and metastatic deposits (intra-patient), or due to differences in retention between different cancer types (inter-tumoural). The mechanisms for the observed heterogeneity are not well understood but likely reflect the inherent molecular differences in tumour subtypes and their respective microenvironments. Nevertheless, these factors limit the utility of ^18^F-FSPG as a diagnostic modality.

In a series of five patients with intracranial tumours, Mittra et al. [36] found that there was significantly less ^18^F-FSPG retention in a low-grade oligodendroglioma, where the blood–brain barrier was more likely to be intact, compared to higher ^18^F-FSPG retention in both a high-grade primary brain cancer (glioblastoma multiforme) and three patients with brain metastases secondary to NSCLC. Also evaluating intracranial disease, Wardak et al. [32] described differences in the ^18^F-FSPG retention time-activity curves of lesions with good vs. poor outcomes in primary brain tumours, finding that high ^18^F-FSPG retention, associated with steeper time-activity curves, corresponded to poor outcomes. Lesion outcome analysis was determined by follow-up MRI and, where available, histopathology. This combined information was evaluated by three clinicians blinded to the ^18^F-FSPG PET/CT results, and the outcome was classified using a scoring system ranging from one to five. Using multi-time-point PET imaging, relative changes in ^18^F-FSPG brain tumour kinetics were able to accurately predict lesion outcome, with an overall accuracy of 89% in the primary brain tumour group, as determined on follow-up MRI and/or pathologic examination.

The heterogeneity of ^18^F-FSPG retention may also be related to the tumour proliferative index, which can be measured by Ki67 immunohistochemical staining [45,46]. Interestingly, in NHL [14], where the ^18^F-FSPG retention was variable between patients, Ki67 staining for the patient with mantle cell lymphoma with low ^18^F-FSPG retention was 6%, while for the second patient with high ^18^F-FSPG retention (nearly double the comparative ^18^F-FDG retention), Ki67 was 20%. These findings with ^18^F-FSPG may be similar to those which have been established with ^18^F-FDG; it is known that the SUV of ^18^F-FDG rises with increased proliferative activity and biological aggressiveness of the tumour tissue, and SUV was shown to have a statistically significant positive correlation with Ki67 across a variety of subtypes of non-Hodgkin’s lymphoma [47]. Overall, these findings suggest that the degree of ^18^F-FSPG retention may be related to lesion proliferation. This makes sense in the context of the oxidative stress of the tumour, with high-grade, highly proliferative lesions under increased oxidative stress compared to low-grade lesions. Subsequent higher expression of system x_c_^−^ and GSH synthesis leads to a decrease in intracellular cystine (Figure 1), increasing the concentration gradient across the cell membrane, thereby bringing in more cystine in exchange for ^18^F-FSPG efflux.

### 3.6. Further Confounds of Imaging Redox Status

It is important to note that imaging redox status is not without its confounding factors. Probably the most important of these is that system x_c_^−^ is upregulated not only in cancer but also in proinflammatory states. ^18^F-FSPG has high retention in a variety of inflammatory and infectious lesions in humans, such as sarcoidosis [48], which could confound its role in tumour identification or treatment response. ^18^F-FSPG is also known to be taken up in activated T cells and microglia and has been used in mouse models to visualise multiple sclerosis [49] and cerebral ischemia [50]. In cerebral ischemia, the upregulation of system x_c_^−^ is thought to be mediated by the inflammatory response. Domercq et al. [50] showed that following transient middle cerebral artery occlusion in rats, ^18^F-FSPG retention was increased, peaking at days 3-7 post-ischemia insult, and was followed by a progressive decrease in ^18^F-FSPG retention from days 14-28 after reperfusion, suggesting that the upregulation of system x_c_^−^ occurs with elevated oxidative stress. It is also expected that ^18^F-FSPG retention will be increased in chronic conditions of oxidative stress in organs where xCT is expressed. Our group has recently looked at this in the context of chronic liver disease [51] in both mouse models and patients with argininosuccinate lyase deficiency, suggesting that ^18^F-FSPG PET could be used as a novel non-invasive diagnostic tool to assess liver disease. Overall, retention in non-tumour sites reduces the specificity of ^18^F-FSPG and could complicate tumour diagnosis, particularly if the patient is being treated with immunotherapy.

Early attempts have been made to develop second-generation radiotracers for molecular imaging of system x_c_^−^, with the potential for reduced retention in inflammatory cells and enhanced tumour visualisation. Beinat et al. [15] have trialled the novel radiotracer ^18^F-hGTS13-isomer2, finding that in cell cultures, tumour-associated radioactivity was significantly higher for ^18^F-hGTS13 than for ^18^F-FSPG and that ^18^F-hGTS13-isomer2 exhibited excellent tumour visualisation with reduced retention in multiple immune cell populations relative to ^18^F-FSPG. However, there was an increase in ^18^F-hGTS13 retention in the liver compared with ^18^F-FSPG, which could limit the success of this radiotracer in the assessment of primary and metastatic liver disease. New research suggests that radiotracer stereochemistry is important. The specificity of the novel radiotracer ^18^F-FRPG for system x_C_^−^ has been tested using transporter inhibition and blocking studies with system x_C_^−^ substrates, finding that ^18^F-FRPG was specifically transported across the plasma membrane by the cystine/glutamate antiporter system x_c_^−^ and retained at high levels in multiple tumour models [16].

## 4. Future Directions

One further avenue of investigation is the use of ^18^F-FSPG as a non-invasive modality for monitoring early treatment response. A major difficulty with non-invasive assessment of treatment response is the timing of when cell death occurs [52]. Molecular imaging agents offer the possibility of measuring tumour cell death prior to quantitative changes in tumour size. However, clinical trials directed at imaging apoptosis by PET, SPECT, and MRI have failed to reliably predict response to treatment with high sensitivity and specificity [53]. One advantage of imaging with ^18^F-FSPG is that changes in the redox status precede cell death, implying that there may be an earlier time frame for assessment of treatment response.

Furthermore, ^18^F-FSPG could be used to identify patients with drug-resistant tumours. Currently, there is no optimal way to identify patients who are refractory to the standard-of-care treatment, and lack of response to first-line treatment or the development of resistance are leading causes of treatment failure in cancer patients. If the preclinical findings showing ^18^F-FSPG that can detect upregulated antioxidant pathways in treatment-resistant cancer [26] could be translated to humans, imaging with ^18^F-FSPG might enable the identification of patients that are primarily refractory or develop resistance to standard-of-care therapy over the course of treatment. Identification of therapy resistance would allow patients with treatment-resistant tumours to switch from standard-of-care treatment to alternative therapies, thereby potentially improving outcomes and avoiding the unnecessary morbidity associated with an ineffective cancer treatment. No studies using ^18^F-FSPG PET/CT as a method to predict treatment resistance in humans, however, have yet been performed.

## 5. Conclusions

Tumour redox status at a cellular level is increasingly well-understood, and targeting the system x_c_^−^ with imaging has provided valuable results in both animal models and humans. All studies to date that have investigated the use of ^18^F-FSPG in humans have been performed on a small scale and focused on the performance of ^18^F-FSPG as a general oncologic diagnostic tracer. The diagnostic performance of this tracer has, in some instances, been hampered by variable tumour retention. The heterogeneity of ^18^F-FSPG retention likely relates to the variable activity of system x_c_^−^, the tumour redox status, and the grade of the tumour. To confirm factors that mediate ^18^F-FSPG retention, multiple further steps are needed: (1) improved immunohistochemical markers of xCT expression; (2) larger-scale studies, which could stratify treatment in patients with tumours with high ^18^F-FSPG retention versus low ^18^F-FSPG retention; and (3) Studies which include both high- and low-grade subtypes of the same cancer. Future uses of ^18^F-FSPG may exploit variable retention between and within cancer types, moving from a diagnostic to a predictive marker for treatment resistance and early response assessment. Promising early preclinical results indicate that changes in ^18^F-FSPG following treatment precede changes in ^18^F-FDG, the current standard-of-care imaging, offer exciting routes forward for cancer care.

## Figures and Tables

**Table 1:** Summary of the clinical ^18^F-FSPG papers published to date.

Reference	Patients	Year	Location	Comparison Imaging	^18^F-FSPG Primary Tumour-to- Background Ratio *	Histo- Pathology
Wardak M. et al. [32]	A total of 26 intracranial malignancies: 17 primary; 7 metastases.	2022	USA	MRI (^18^F-FDG PET/CT in 4)	26.6 ± 24.9	xCT in 19/26
Park S. Y. et al. [33]	A total of 20 prostate cancers: 10 primary; 10 recurrent.	2020	USA	Up to five lesions per patient were selected and measured on MRI, CT, or bone scan.	2.0 ± 0.5	xCT and CD44
Park S. Y. et al. [14]	A total of 15 patients: 5 head and neck; 5 Non-Hodgkin’s lymphoma; 5 Colorectal.	2020	USA	^18^F-FDG PET/CT	Head and neck: 2.8 ± 2.1 Non-Hodgkin’s lymphoma: 1.9 ± 3.1 Colorectal: 5.4 ± 1.3	No histopathology reference standard.
Cheng M. -F. et al. [34]	A total of 23 patients: all pancreatic adenocarcinoma.	2019	Taiwan	^18^F-FDG PET/CT	4.2 ± 4.3	xCT expression (in 6/23).
Magarik M. A. et al. [31]	Single-case study: NSCLC with mediastinal and intracranial metastases.	2018	USA	^18^F-FDG PET/CT	Not described	No histopathology reference standard.
Kavanaugh G. et al. [35]	A total of 11 patients with HCC.	2016	USA	Standard of care: MRI, CT, and ^11^C-acetate PET/CT	Not described	No histopathology reference standard for the 11 patients. Comparison made to tissue from cancer genome analysis (x_c_^−^ transporter RNA and protein levels).
Mittra E. S. et al. [36]	A total of 5 patients: 2 primary brain tumours; 3 NSCLC with brain metastasis.	2016	USA	^18^F-FDG PET/CT	Primary: 24.0 Metastases 50.0 (background: normal brain).	No histopathology reference standard.
Baek S. et al. [13]	A total of 5 patients with HCC.	2013	South Korea	^18^F-FDG PET/CT	Not described	xCT and CD44 in 4/5
Baek S. et al. [37]	A total of 15 patients: 10 lung cancer; 5 breast cancer.	2012	South Korea	^18^F-FDG PET/CT	Lung: 6.7 ± 5.8 Breast: 3.7 ± 4.5 (background: blood pool)	xCT and CD44-specific antibody
Paez R. et al. [38]	A total of 26 patients with indeterminate pulmonary nodules.	2022	USA	^18^F-FDG PET/CT	Not described	xCT and CD44 expression levels

* SUV_mean_ at 60 min where available.
